# Supplementary material for: Inhibition of EIF2α Dephosphorylation Decreases Cell Viability and Synergizes with Standard-of-Care Chemotherapeutics in Head and Neck Squamous Cell Carcinoma
Source: Cancers (Basel). 2023 Nov 9;15(22):5350. doi: 10.3390/cancers15225350 (PMC10670742; doi:10.3390/cancers15225350)
Supplement: Supplementary file 1 [file cancers-15-05350-s001.zip › cancers-2673541-supplementary.pdf]

Supplementary Table S1. Antibodies used in the study.

Primary antibody		Manufacturer (Cat. No)	Dilution	Species of origin
eIF2**α**	monoclonal	Cell Signaling (#5324)	1:1000 (WB)<br>1:2000 (IHC)	Rabbit
phospho-eIF2**α** (Ser51)	monoclonal	Cell Signaling (#3398)	1:1000 (WB)	Rabbit
GAPDH (14C10)	monoclonal	Cell Signaling (#2118)	1:3000	Rabbit
HPV16/18	monoclonal	Exalpha Biologicals (HP305)<br>Secondary reagents: BIOLOGO Universal Staining System DAB (# DA005)	Ready - use (IHC)	Mouse
p16	monoclonal	Exalpha Biologicals (P16002)<br>Secondary reagents: BIOLOGO Universal Staining System DAB (# DA005)	1:10 (IHC)	Mouse
**α**-Tubulin	monoclonal	SantaCruz sc-5286	1:1000	Mouse
Cyclin A	monoclonal	SantaCruz sc-271682	1:500	Mouse
Cyclin D1	monoclonal	SantaCruz sc-8396	1:500	Mouse
CDK2	monoclonal	SantaCruz sc-6248	1:500	Mouse
E2F1	polyclonal	Cell Signaling (##3742)	1:1000	Rabbit
p21	monoclonal	Cell Signaling (#2947)	1:1000	Rabbit
Rb1	monoclonal	Invitrogen (SY63-03)	1:1000	Rabbit
pRb1 (Ser780)	polyclonal	Invitrogen (PA5-114632)	1:1000	Rabbit
pRb1 (Ser807/811)	monoclonal	Invitrogen (13H27L9)	1:500	Rabbit
Anti-Rabbit	HRP-linked	Cell Signaling (#7074)	1:2000	Goat
Anti-mouse	HRP-linked	Cell Signaling (#7076)	1:2000	Horse

Supplementary Figure 1.1. *EIF2S1* is elevated in HNSCC at all stages and all histological grades in comparison to normal epithelium. In all box-and-whisker diagrams horizontal line within the box corresponds to median, box boundaries to lower and upper quartiles and vertical lines stretch from minimum to maximum values. Statistical analysis was performed by comparing the interquartile range of each group by Welch's t-test, significance level p<0.05.

(A)

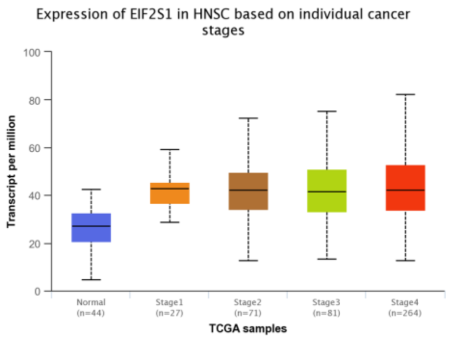

Comparison	Statistical significance
Normal-vs-Stage1	3.15E-07
Normal-vs-Stage2	1.46E-08
Normal-vs-Stage3	2.67E-12
Normal-vs-Stage4	1.62E-12
Stage1-vs-Stage2	4.31E-01
Stage1-vs-Stage3	6.20E-01
Stage1-vs-Stage4	9.74E-01
Stage2-vs-Stage3	1.77E-01
Stage2-vs-Stage4	3.14E-01
Stage3-vs-Stage4	4.47E-01

(B)

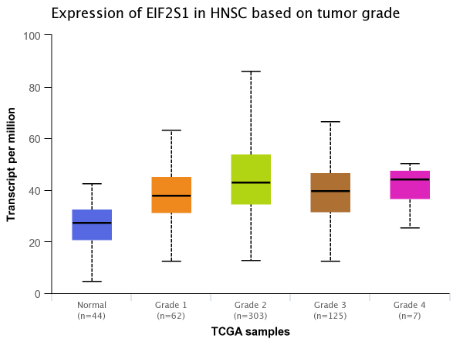

Comparison	Statistical significance
Normal-vs-Grade 1	9.04E-10
Normal-vs-Grade 2	1.62E-12
Normal-vs-Grade 3	1.20E-13
Normal-vs-Grade 4	9.77E-05
Grade 1-vs-Grade 2	1.26E-03
Grade 1-vs-Grade 3	7.24E-01
Grade 1-vs-Grade 4	9.12E-01
Grade 2-vs-Grade 3	6.25E-04
Grade 2-vs-Grade 4	1.34E-01
Grade 3-vs-Grade 4	9.72E-01

Supplementary Figure 1.2. *EIF2S2* and *EIF2S3* genes are overexpressed in head and neck squamous cell carcinoma (HNSCC). **(A, B)** *EIF2S2* and *EIF2S3* transcript expression are elevated in HNSCC of all stages, the increase becomes more pronounced with cancer progression. **(C, D)** *EIF2S2* and *EIF2S3* are elevated in HNSCC across all histological grades. The transcript expression increases with increasing cancer grade. In all box-and-whisker diagrams horizontal line within the box corresponds to median, box boundaries to lower and upper quartiles and vertical lines stretch from minimum to maximum values. Statistical analysis was performed by comparing the interquartile range of each group by Welch's t-test, significance level $p<0.05$.

(A)

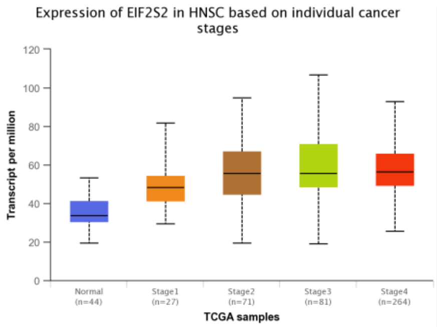

Comparison	Statistical significance
Normal-vs-Stage1	4.96E-03
Normal-vs-Stage2	2.44E-08
Normal-vs-Stage3	8.52E-10
Normal-vs-Stage4	1.62E-12
Stage1-vs-Stage2	7.87E-01
Stage1-vs-Stage3	3.31E-01
Stage1-vs-Stage4	7.27E-01
Stage2-vs-Stage3	2.82E-01
Stage2-vs-Stage4	9.04E-01
Stage3-vs-Stage4	1.98E-01

(B)

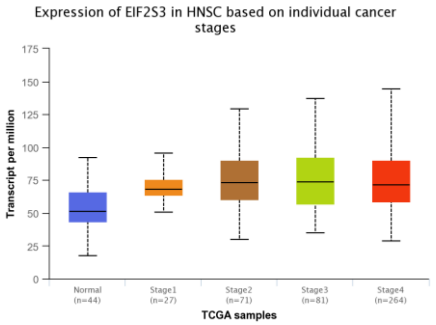

Comparison	Statistical significance
Normal-vs-Stage1	1.91E-02
Normal-vs-Stage2	5.55E-09
Normal-vs-Stage3	9.03E-08
Normal-vs-Stage4	8.46E-13
Stage1-vs-Stage2	7.76E-01
Stage1-vs-Stage3	7.77E-01
Stage1-vs-Stage4	8.25E-01
Stage2-vs-Stage3	9.95E-01
Stage2-vs-Stage4	8.41E-01
Stage3-vs-Stage4	8.35E-01

(C)

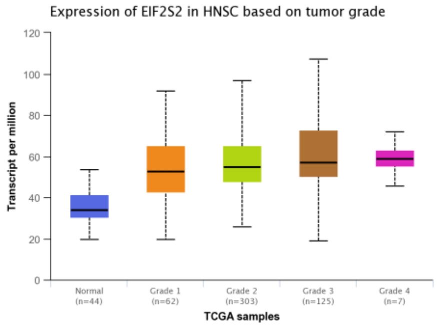

Comparison	Statistical significance
Normal-vs-Grade 1	8.70E-08
Normal-vs-Grade 2	1.62E-12
Normal-vs-Grade 3	<1E-12
Normal-vs-Grade 4	2.68E-09
Grade 1-vs-Grade 2	3.87E-01
Grade 1-vs-Grade 3	5.33E-02
Grade 1-vs-Grade 4	8.72E-01
Grade 2-vs-Grade 3	6.40E-02
Grade 2-vs-Grade 4	4.65E-01
Grade 3-vs-Grade 4	4.76E-02

(D)

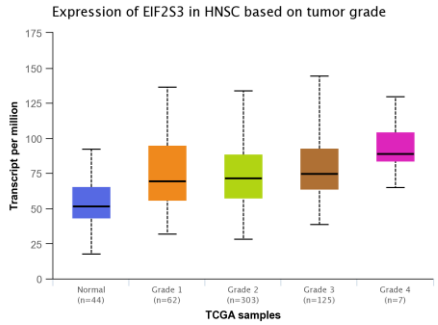

Comparison	Statistical significance
Normal-vs-Grade 1	1.63E-05
Normal-vs-Grade 2	2.42E-12
Normal-vs-Grade 3	6.66E-12
Normal-vs-Grade 4	2.13E-02
Grade 1-vs-Grade 2	2.86E-01
Grade 1-vs-Grade 3	1.96E-02
Grade 1-vs-Grade 4	9.53E-02
Grade 2-vs-Grade 3	8.20E-02
Grade 2-vs-Grade 4	1.37E-02
Grade 3-vs-Grade 4	1.13E-01

Supplementary Figure 2.1. *EIF2B1* and *EIF2B2* genes are overexpressed in head and neck squamous cell carcinoma (HNSCC). **(A, B)** *EIF2B1* and *EIF2B2* transcript expression is elevated in HNSCC of all stages, the increase becomes more pronounced with cancer progression. **(C, D)** *EIF2B1* and *EIF2B2* is elevated in HNSCC across all histological grades. *EIF2B1* transcript expression increases with increasing cancer grade, whereas *EIF2B2* is highest in grade 2 tumors. In all box-and-whisker diagrams horizontal line within the box corresponds to median, box boundaries to lower and upper quartiles and vertical continuous lines stretch from minimum to maximum values. Statistical analysis was performed by comparing the interquartile range of each group by Welch's t-test, significance level $p<0.05$.

(A)

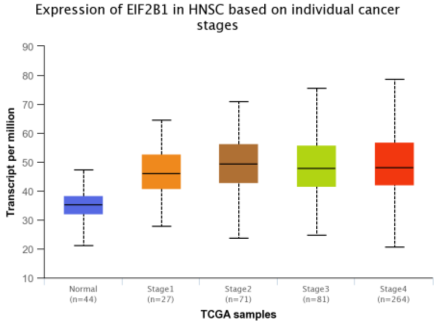

Comparison	Statistical significance
Normal-vs-Stage1	1.70E-05
Normal-vs-Stage2	2.36E-13
Normal-vs-Stage3	1.94E-13
Normal-vs-Stage4	1.62E-12
Stage1-vs-Stage2	5.49E-01
Stage1-vs-Stage3	7.02E-01
Stage1-vs-Stage4	5.22E-01
Stage2-vs-Stage3	7.94E-01
Stage2-vs-Stage4	8.01E-01
Stage3-vs-Stage4	5.91E-01

(C)

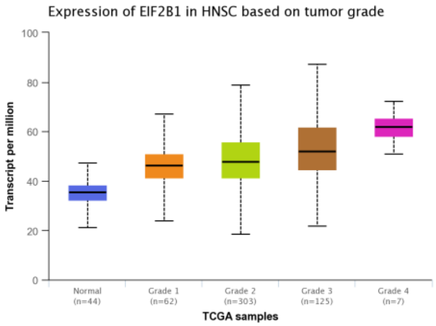

Comparison	Statistical significance
Normal-vs-Grade 1	4.19E-08
Normal-vs-Grade 2	1.62E-12
Normal-vs-Grade 3	<1E-12
Normal-vs-Grade 4	5.28E-11
Grade 1-vs-Grade 2	7.17E-02
Grade 1-vs-Grade 3	7.44E-04
Grade 1-vs-Grade 4	2.66E-03
Grade 2-vs-Grade 3	2.38E-02
Grade 2-vs-Grade 4	3.64E-02
Grade 3-vs-Grade 4	1.14E-01

(B)

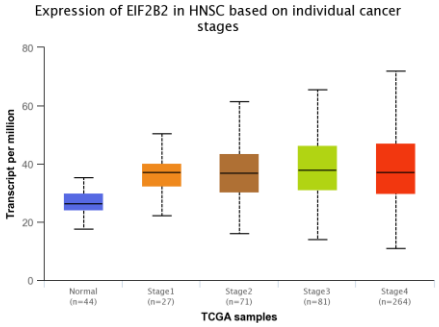

Comparison	Statistical significance
Normal-vs-Stage1	1.79E-05
Normal-vs-Stage2	1.70E-08
Normal-vs-Stage3	1.88E-12
Normal-vs-Stage4	1.62E-12
Stage1-vs-Stage2	5.93E-01
Stage1-vs-Stage3	8.22E-01
Stage1-vs-Stage4	9.75E-01
Stage2-vs-Stage3	6.76E-01
Stage2-vs-Stage4	4.88E-01
Stage3-vs-Stage4	7.44E-01

(D)

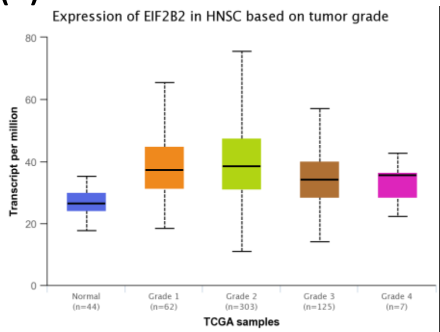

Comparison	Statistical significance
Normal-vs-Grade 1	9.01E-11
Normal-vs-Grade 2	1.62E-12
Normal-vs-Grade 3	8.87E-12
Normal-vs-Grade 4	9.08E-03
Grade 1-vs-Grade 2	1.86E-01
Grade 1-vs-Grade 3	9.13E-02
Grade 1-vs-Grade 4	1.75E-01
Grade 2-vs-Grade 3	6.34E-05
Grade 2-vs-Grade 4	1.18E-02
Grade 3-vs-Grade 4	4.40E-01

Supplementary Figure 2.2. *EIF2B3* gene is overexpressed in head and neck squamous cell carcinoma (HNSCC). **(A and C)** *EIF2B3* transcript expression is elevated in HNSCC of all stages and grades, the increase becomes more pronounced with cancer progression. **(B and D)** *EIF2B4* transcript is unchanged across tumor stages and grades. In all box-and-whisker diagrams horizontal line within the box corresponds to median, box boundaries to lower and upper quartiles and vertical lines stretch from minimum to maximum values. Statistical analysis was performed by comparing the interquartile range of each group by Welch's t-test, significance level $p<0.05$.

(A)

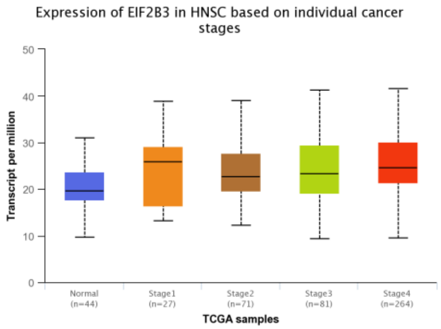

Comparison	Statistical significance
Normal-vs-Stage1	4.20E-02
Normal-vs-Stage2	1.80E-03
Normal-vs-Stage3	2.04E-03
Normal-vs-Stage4	7.50E-08
Stage1-vs-Stage2	6.33E-01
Stage1-vs-Stage3	7.36E-01
Stage1-vs-Stage4	1.27E-01
Stage2-vs-Stage3	8.26E-01
Stage2-vs-Stage4	1.19E-01
Stage3-vs-Stage4	5.49E-02

(B)

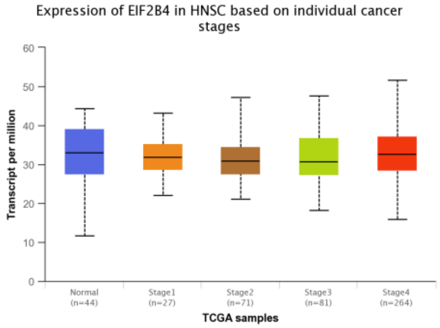

Comparison	Statistical significance
Normal-vs-Stage1	5.75E-01
Normal-vs-Stage2	8.41E-01
Normal-vs-Stage3	7.65E-01
Normal-vs-Stage4	3.59E-01
Stage1-vs-Stage2	7.10E-01
Stage1-vs-Stage3	7.38E-01
Stage1-vs-Stage4	9.09E-01
Stage2-vs-Stage3	9.29E-01
Stage2-vs-Stage4	4.14E-01
Stage3-vs-Stage4	4.48E-01

(C)

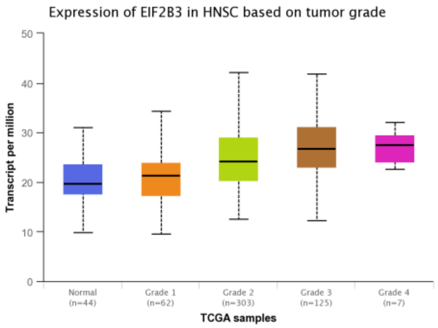

Comparison	Statistical significance
Normal-vs-Grade 1	1.35E-01
Normal-vs-Grade 2	2.10E-06
Normal-vs-Grade 3	3.82E-09
Normal-vs-Grade 4	3.98E-04
Grade 1-vs-Grade 2	1.05E-02
Grade 1-vs-Grade 3	4.20E-05
Grade 1-vs-Grade 4	1.53E-02
Grade 2-vs-Grade 3	1.42E-02
Grade 2-vs-Grade 4	1.78E-01
Grade 3-vs-Grade 4	4.98E-01

(D)

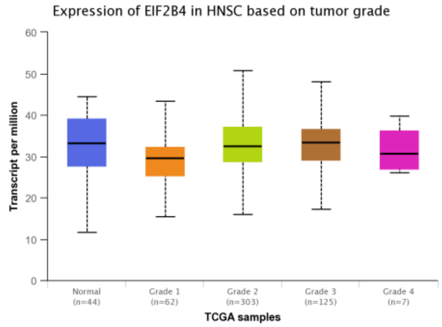

Comparison	Statistical significance
Normal-vs-Grade 1	6.01E-01
Normal-vs-Grade 2	3.15E-01
Normal-vs-Grade 3	4.07E-01
Normal-vs-Grade 4	7.40E-01
Grade 1-vs-Grade 2	6.22E-02
Grade 1-vs-Grade 3	1.22E-01
Grade 1-vs-Grade 4	9.85E-01
Grade 2-vs-Grade 3	7.77E-01
Grade 2-vs-Grade 4	4.74E-01
Grade 3-vs-Grade 4	4.89E-01

Supplementary Figure 2.3. *EIF2B5* gene is overexpressed in head and neck squamous cell carcinoma (HNSCC). **(A)** *EIF2B5* transcript expression is elevated in HNSCC at stages 2, 3 and 4, the increase becomes more pronounced with cancer progression. **(B)** *EIF2B5* is elevated in HNSCC across all histological grades, the median number of transcripts increases gradually from Grade 1 to 4. In all box-and-whisker diagrams horizontal line within the box corresponds to median, box boundaries to lower and upper quartiles and continuous lines stretch from minimum to maximum values. Statistical analysis was performed by comparing the interquartile range of each group by Welch's t-test, significance level $p<0.05$.

(A)

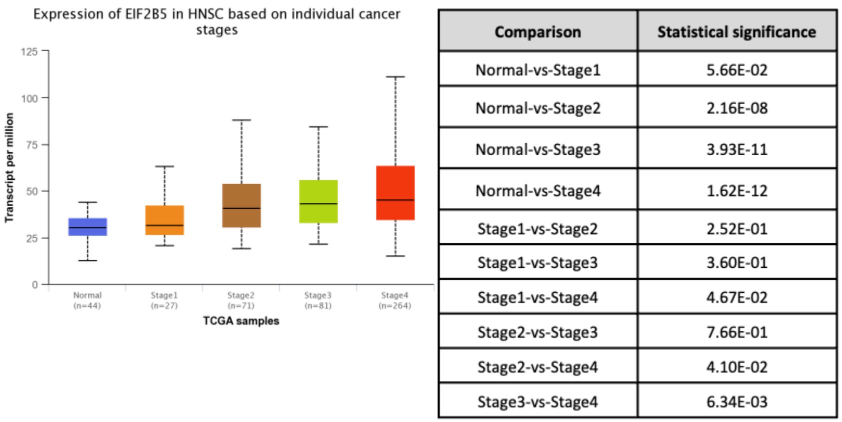

Comparison	Statistical significance
Normal-vs-Stage1	5.66E-02
Normal-vs-Stage2	2.16E-08
Normal-vs-Stage3	3.93E-11
Normal-vs-Stage4	1.62E-12
Stage1-vs-Stage2	2.52E-01
Stage1-vs-Stage3	3.60E-01
Stage1-vs-Stage4	4.67E-02
Stage2-vs-Stage3	7.66E-01
Stage2-vs-Stage4	4.10E-02
Stage3-vs-Stage4	6.34E-03

(B)

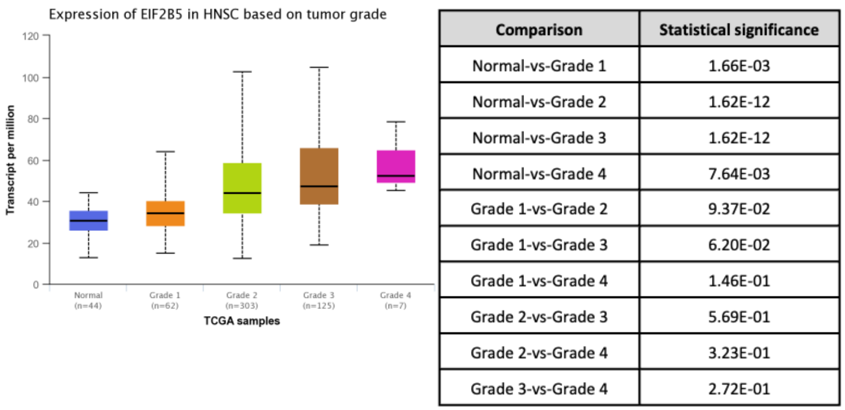

Comparison	Statistical significance
Normal-vs-Grade 1	1.66E-03
Normal-vs-Grade 2	1.62E-12
Normal-vs-Grade 3	1.62E-12
Normal-vs-Grade 4	7.64E-03
Grade 1-vs-Grade 2	9.37E-02
Grade 1-vs-Grade 3	6.20E-02
Grade 1-vs-Grade 4	1.46E-01
Grade 2-vs-Grade 3	5.69E-01
Grade 2-vs-Grade 4	3.23E-01
Grade 3-vs-Grade 4	2.72E-01

Supplementary Figure 3. *EIF2S1* promoter methylation levels are decreased in head and neck carcinoma (HNSCC). Beta values indicate the degree of promoter methylation ranging from 0 – unmethylated to 1- methylated. Beta values are obtained by dividing the methylated probe intensity by the total probe intensity (methylated and unmethylated). **(A)** Stage 2, 3 and 4 HNSCC show a lower degree of *EIF2S1* promoter methylation than normal samples. **(B)** Tumors with mutated *P53* have lower promoter methylation than non-mutant and normal samples. Statistical significance was determined with student t-test, significance level $p<0.05$.

(A)

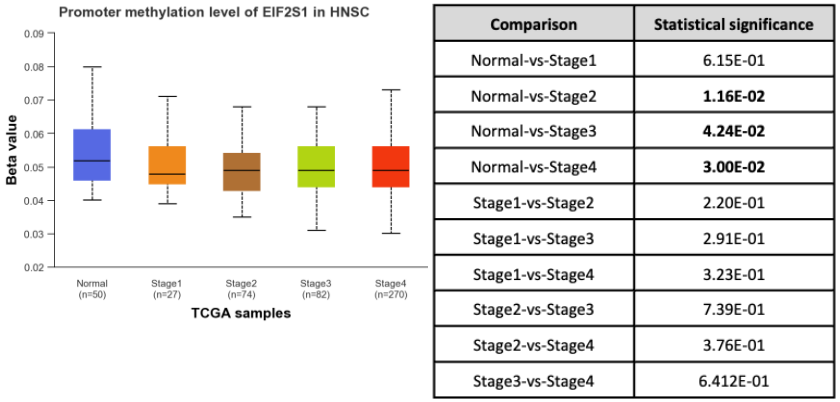

Comparison	Statistical significance
Normal-vs-Stage1	6.15E-01
Normal-vs-Stage2	**1.16E-02**
Normal-vs-Stage3	**4.24E-02**
Normal-vs-Stage4	**3.00E-02**
Stage1-vs-Stage2	2.20E-01
Stage1-vs-Stage3	2.91E-01
Stage1-vs-Stage4	3.23E-01
Stage2-vs-Stage3	7.39E-01
Stage2-vs-Stage4	3.76E-01
Stage3-vs-Stage4	6.412E-01

(B)

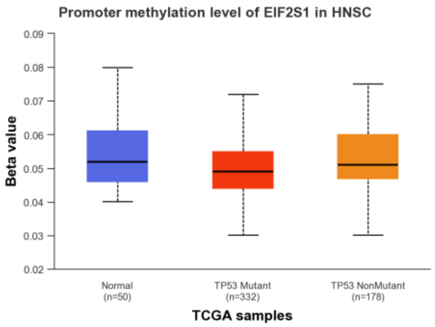

Comparison	Statistical significance
Normal-vs-TP53 Mutant	**7.24E-03**
Normal-vs-TP53 NonMutant	8.78E-01
TP53 Mutant-vs-TP53 NonMutant	**6.25E-05**

Supplementary Figure 4.1. EIF2α and EIF2β (encoded by *EIF2S1* and *EIF2S2*) are overexpressed in HNSCC. **(A, B)** EIF2α and EIF2β are elevated in HNSCC, the overexpression becomes more pronounced with cancer stage. **(C, D)** EIF2α and EIF2β expression is elevated on all histological grades and highest in grade 2 HNSCC. In all box-and-whisker diagrams horizontal line within the box corresponds to median, box boundaries to lower and upper quartiles and vertical continuous lines stretch from minimum to maximum values. Statistical analysis was performed by comparing the interquartile range of each group by Welch's t-test, significance level $p<0.05$.

(A)

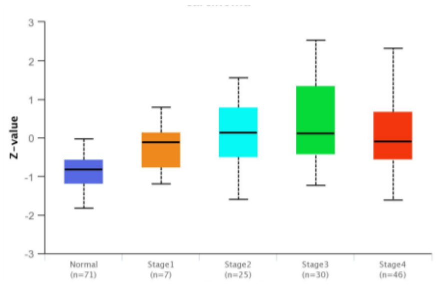

Comparison	Statistical significance
Normal-vs-Stage1	6.93E-02
Normal-vs-Stage2	8.85E-05
Normal-vs-Stage3	1.29E-06
Normal-vs-Stage4	9.25E-08
Stage1-vs-Stage2	3.05E-01
Stage1-vs-Stage3	5.02E-02
Stage1-vs-Stage4	3.45E-01
Stage2-vs-Stage3	2.06E-01
Stage2-vs-Stage4	8.14E-01
Stage3-vs-Stage4	9.63E-02

(B)

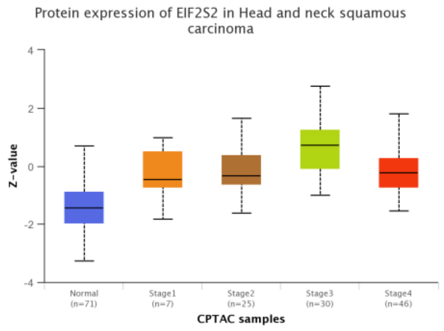

Comparison	Statistical significance
Normal-vs-Stage1	3.14E-02
Normal-vs-Stage2	1.12E-06
Normal-vs-Stage3	3.07E-12
Normal-vs-Stage4	4.03E-10
Stage1-vs-Stage2	6.34E-01
Stage1-vs-Stage3	5.85E-02
Stage1-vs-Stage4	5.64E-01
Stage2-vs-Stage3	1.13E-02
Stage2-vs-Stage4	8.83E-01
Stage3-vs-Stage4	6.99E-03

(C)

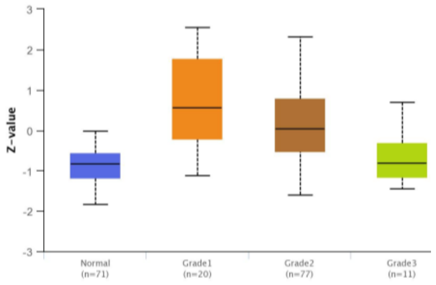

Comparison	Statistical significance
Normal-vs-Grade1	6.00E-06
Normal-vs-Grade2	3.66E-13
Normal-vs-Grade3	5.06E-01
Grade1-vs-Grade2	8.67E-05
Grade1-vs-Grade3	1.81E-03
Grade2-vs-Grade3	<1E-12

(D)

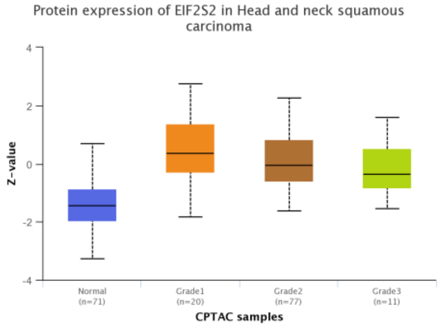

Comparison	Statistical significance
Normal-vs-Grade1	2.88E-06
Normal-vs-Grade2	3.96E-16
Normal-vs-Grade3	3.61E-03
Grade1-vs-Grade2	8.77E-02
Grade1-vs-Grade3	3.75E-01
Grade2-vs-Grade3	<1E-12

Supplementary Figure 4.2. *EIF2S3* gene product EIF2γ is overexpressed in head and neck squamous cell carcinoma (HNSCC). EIF2γ protein abundance is elevated in HNSCC of all stages, the increase becomes more pronounced with cancer progression. EIF2γ are elevated in HNSCC of all histological grades and highest in grade 1 tumors. In all box-and-whisker diagrams horizontal line within the box corresponds to median, box boundaries to lower and upper quartiles and vertical continuous lines stretch from minimum to maximum values. Statistical analysis was performed by comparing the interquartile range of each group by Welch's t-test, significance level $p<0.05$.

(A)

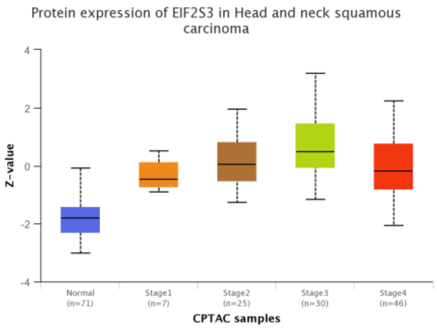

Comparison	Statistical significance
Normal-vs-Stage1	1.49E-04
Normal-vs-Stage2	1.25E-11
Normal-vs-Stage3	3.07E-14
Normal-vs-Stage4	2.72E-16
Stage1-vs-Stage2	1.36E-01
Stage1-vs-Stage3	4.96E-03
Stage1-vs-Stage4	3.62E-01
Stage2-vs-Stage3	7.24E-02
Stage2-vs-Stage4	3.10E-01
Stage3-vs-Stage4	6.70E-03

(B)

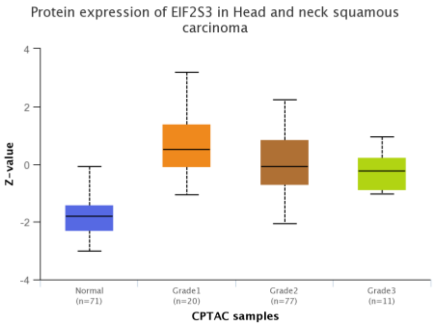

Comparison	Statistical significance
Normal-vs-Grade1	1.14E-09
Normal-vs-Grade2	4.07E-26
Normal-vs-Grade3	4.97E-06
Grade1-vs-Grade2	7.44E-03
Grade1-vs-Grade3	2.09E-01
Grade2-vs-Grade3	<1E-12

Supplementary Figure 5. Heatmap illustrating statistical significance levels of differences between the total immunostaining score (TIS) **(A)** and the intensity score (IS) **(B)** values between different types of histological lesions: metastasis (META); carcinoma (CA); carcinoma *in situ* (CIS); dysplasia (D) and non-neoplastic mucosa (NNPL). P-values were obtained by X^2-test.

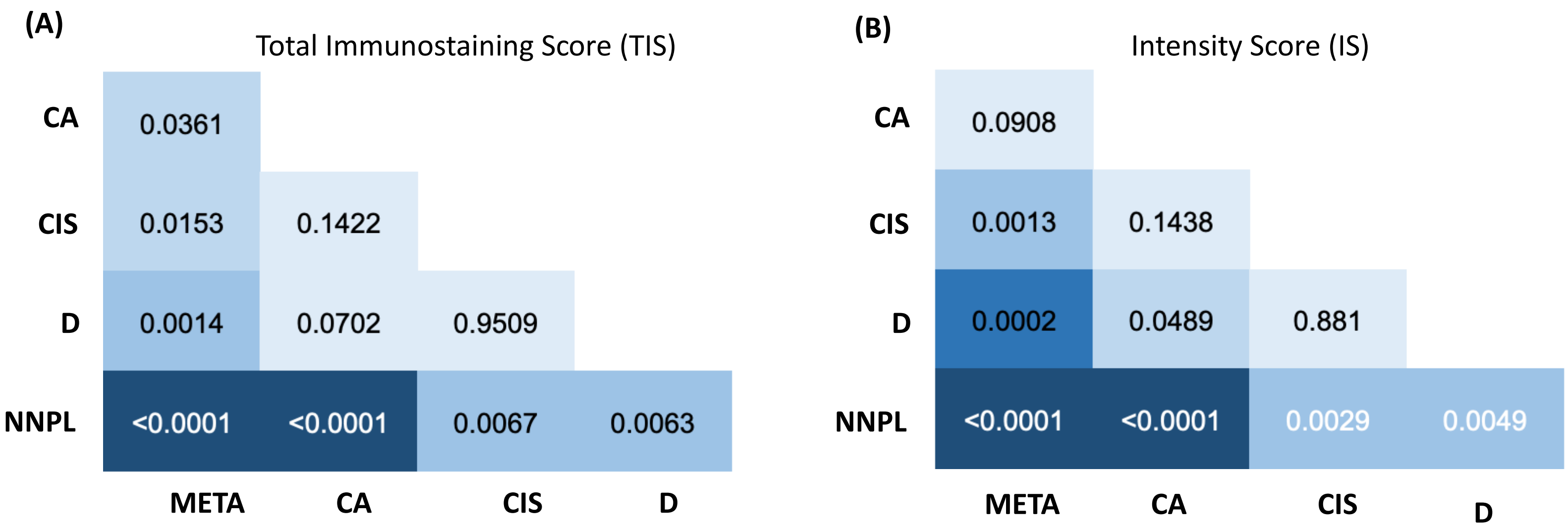

Supplementary Figure 6. Original, uncropped immunoblots from cryosamples. **(A)** Phospho-EIF2α (36 kDa). **(B)** EIF2α (36kDa). **(C)** GAPDH (36 kDa).

(A)

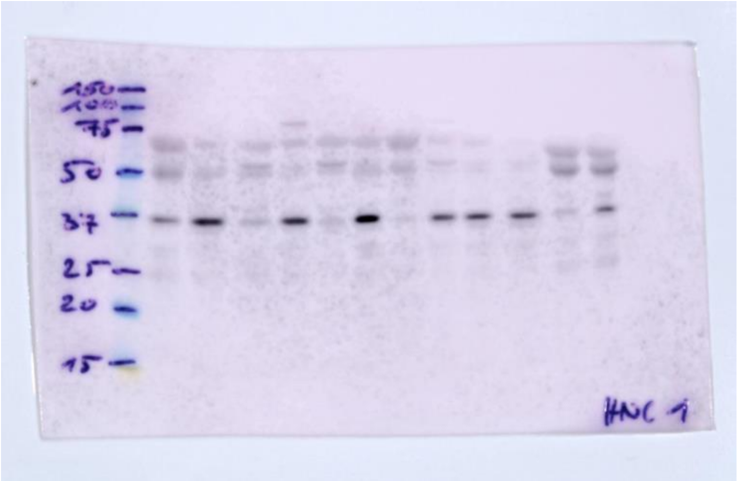

(B)

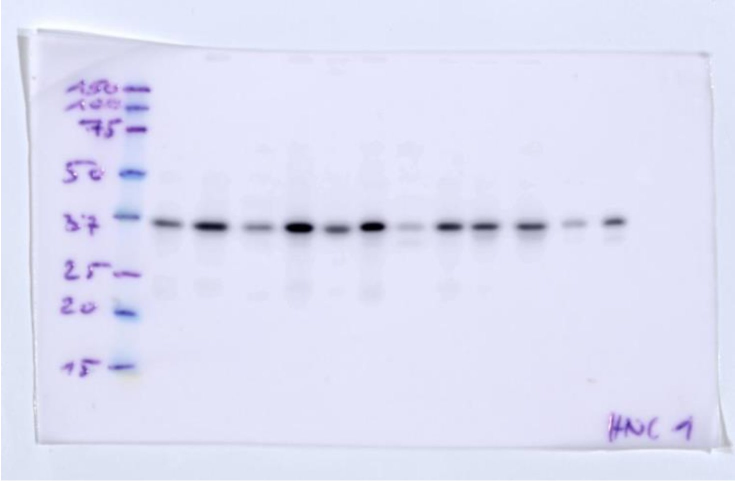

(C)

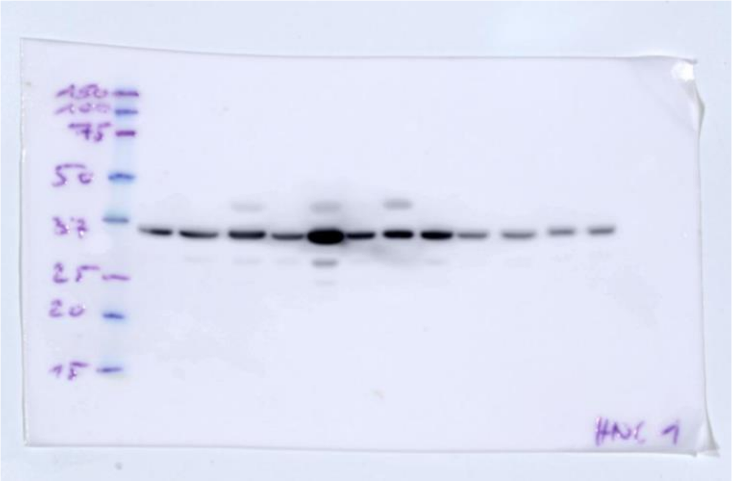

Supplementary Figure 7. (A) *EIF2S1* expression differs with HPV and *P53* mutation status in head and neck carcinoma (HNSCC). *EIF2S1* transcript expression is highest in HPV-negative tumors. In HPV+ positive HNSCC *EIF2S1* is also increased when compared to normal control. Statistical significance was determined with student t-test, significance level $p<0.05$. Data source: TCGA (HNSCC n= 520; normal control n=44), accessed via ualcan.path.uab.edu. **(B)** Immunohistochemistry of HNSCC tissue samples shows a trend for higher eIF2α expression in HPV- and P16- negative HNSCC (p-value: ns; n=83).

(A)

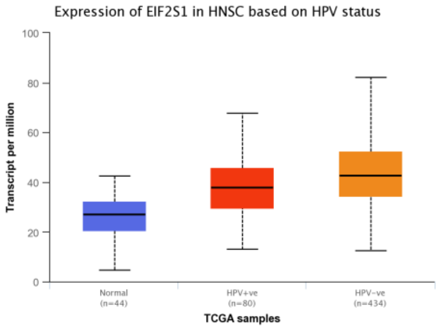

Comparison	Statistical significance
Normal-vs-HPV+ve	2.53E-09
Normal-vs-HPV-ve	<1E-12
HPV+ve-vs-HPV-ve	1.36E-03

(B)

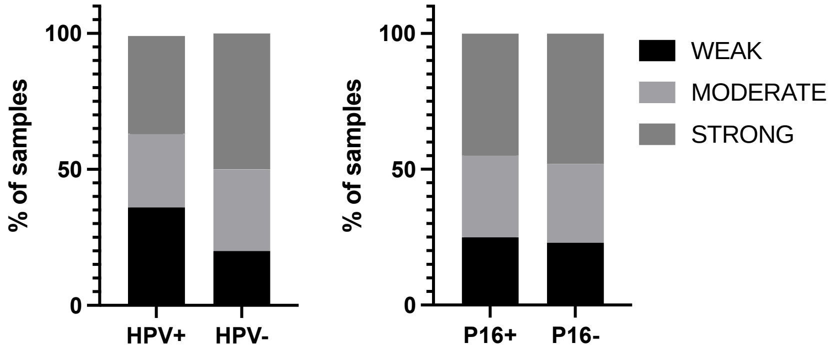

Supplementary Figure 8. Immunoblots and densitometric analysis of EIF2α and phospho-EIF2α abundance after 12h treatment with salubrinal (non-treated, 10, 20 and 50 μM) in FaDu and SCC4 cells.

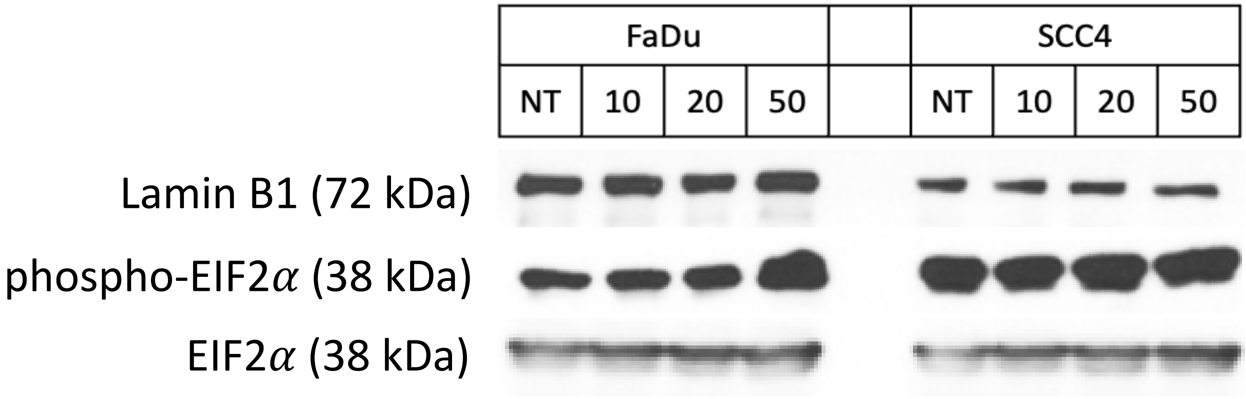

Densitometric analysis		phospho-eIF2a:eIF2a
FaDu	NT	1
	10	1.19584212
	20	1.279310135
	50	1.85494508
SCC4	NT	1
	10	1.208618336
	20	1.213790672
	50	1.129933483

Supplementary Figure 9. The effects of gene ablation and dependency scores of eIF2 complex members, eIF2 kinases and selected phosphatases across 70 HNSCC cell lines. Publicly available data from CRISPR-Cas9 knockdown experiments provided by the DepMap Portal (www.depmap.org) were analyzed and are represented as two measures. **(A)** The Dependency Score depicts the degree to which a given gene is essential for cell survival and proliferation. A dependency score of 0 indicates that a gene is not essential; a score of 1 corresponds to the median of all pan-essential genes. EIF2 complex genes are common essential genes across HNSCC cell lines, in contrast, kinases (EIF2AK1-4) and phosphatases PPP1R15A (encoding GADD34) and PPP1R15B (encoding CREP) are not. **(B)** The CRISPR Gene Effect describes the perturbation effects of the ablation a given gene in cells. Scores close to 0 indicate non-essential genes, while negative scores indicate a higher likelihood that the gene of interest is essential.

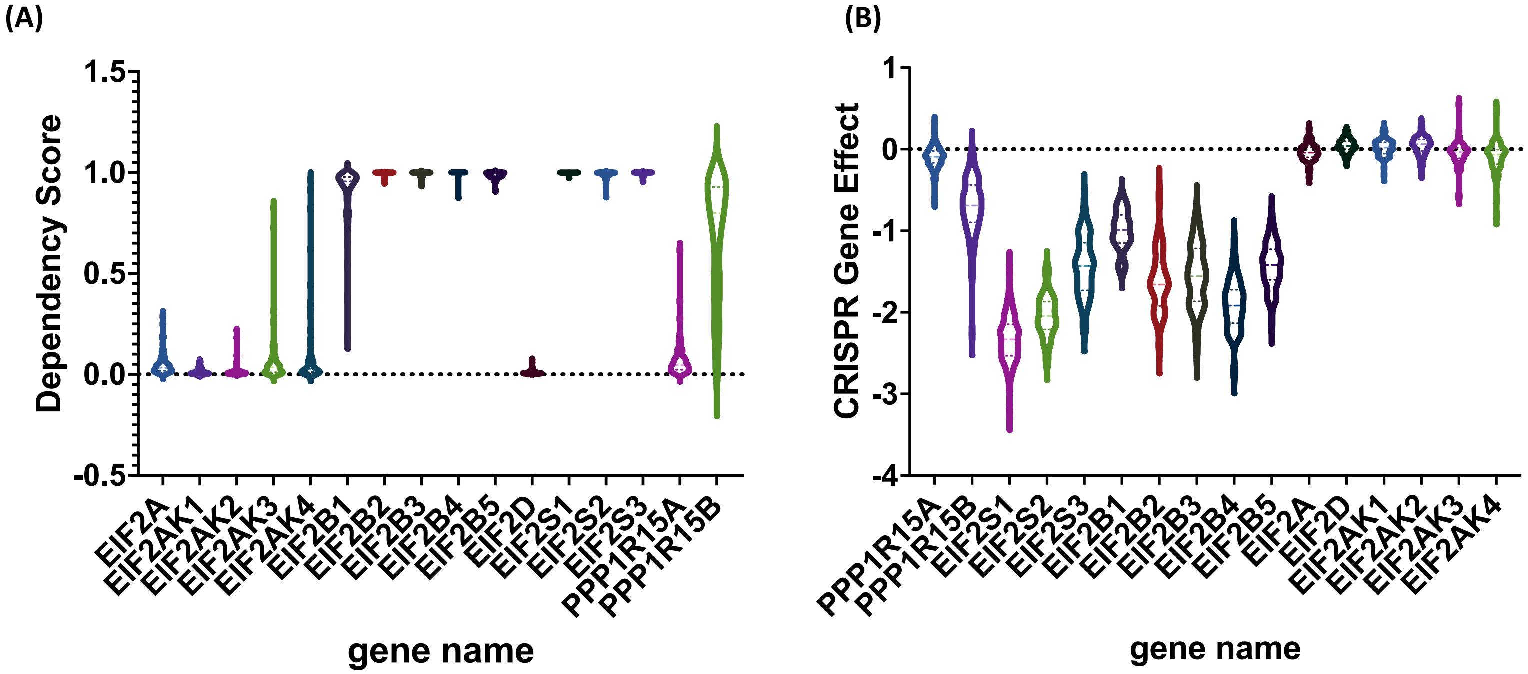

Supplementary Material 10. Original uncropped immunoblot images and densitometric analysis (continued on following pages).

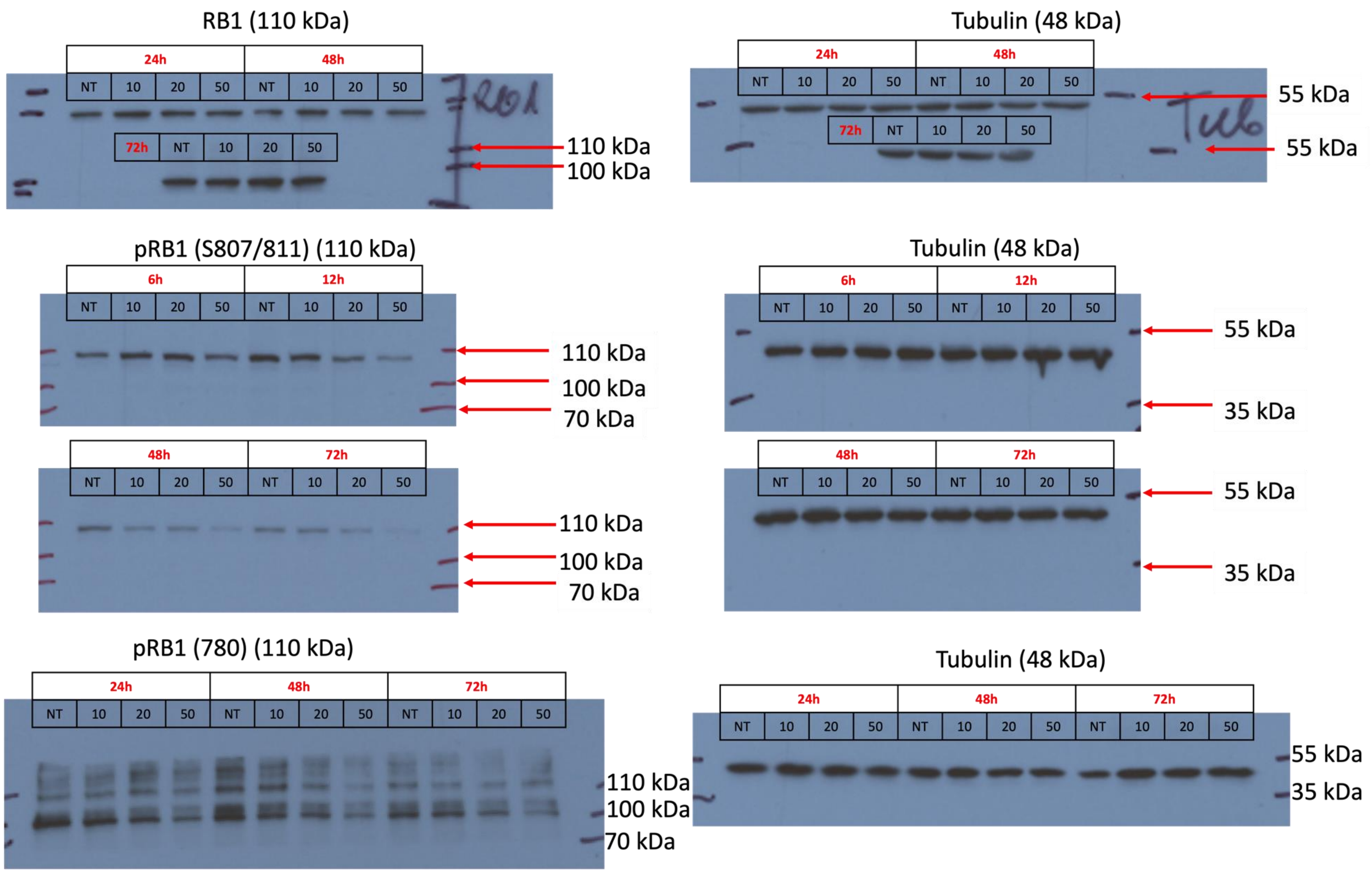

	RB1			normalized - tubulin
12h	NT	18281.915	1	1
	10	19026.329	1.0407186	1.00299327
	20	24757.371	1.35420009	1.37787475
	50	21346.886	1.16765043	1.02814504
24h	NT	8479.853	1	1
	10	15694.723	1.85082489	1.83542887
	20	11909.581	1.40445607	1.96115398
	50	11029.217	1.30063776	1.49700466
72h	NT	6853.903	1	1
	10	11660.995	1.70136563	1.47895513
	20	9570.51	1.39635913	1.50575698
	50	9757.752	1.42367816	1.75709369

	Tubulin for RB1		
12h	NT	6809.447	1
	10	7065.569	1.03761275
	20	6692.447	0.98281799
	50	7733.397	1.1356865
24h	NT	8141.518	1
	10	8209.811	1.00838824
	20	5830.447	0.71613758
	50	7073.569	0.8688268
72h	NT	8795.225	1
	10	10117.882	1.15038353
	20	8156.225	0.92734694
	50	7126.296	0.81024601

	pRB1 (807/811)			normalized - tubulin
12h	NT	34838.413	1	1
	10	33657.927	0.96611539	0.99826244
	20	24235.472	0.69565373	1.01330247
	50	15140.945	0.4346049	0.99967121
24h	NT	19551.622	1	1
	10	11589.53	0.59276565	0.99711047
	20	13077.187	0.66885433	1.00821872
	50	6043.681	0.30911405	1.00796556
72h	NT	15260.551	1	1
	10	15936.915	1.04432107	1.04506873
	20	9164.874	0.60055984	1.03780298
	50	2561.347	0.16784106	1.02077908

	Tubulin for pRB1 (807/811)		
12h	NT	103930.806	1
	10	103750.22	0.99826244
	20	105313.342	1.01330247
	50	103896.635	0.99967121
24h	NT	102700.978	1
	10	102404.22	0.99711047
	20	103545.049	1.00821872
	50	103519.049	1.00796556
72h	NT	99531.463	1
	10	104017.22	1.04506873
	20	103294.049	1.03780298
	50	101599.635	1.02077908

	pRB1 (780)			normalized - tubulin
12h	NT	15758.53	1	1
	10	12350.945	0.78376251	0.67465576
	20	8991.631	0.57058818	0.53518512
	50	5032.418	0.31934565	0.36845895
24h	NT	15285.702	1	1
	10	9944.924	0.65060303	0.64787285
	20	7552.681	0.49410102	0.56869988
	50	3655.175	0.23912379	0.2937009
72h	NT	9573.167	1	1
	10	9416.045	0.98358725	0.49775874
	20	5188.66	0.54200036	0.30619843
	50	3754.589	0.39219926	0.18715051

	Tubulin for pRB1 (780)		
12h	NT	9747.054	1
	10	11323.368	1.1617221
	20	10391.832	1.06615106
	50	8447.832	0.86670619
24h	NT	8641.054	1
	10	8677.468	1.00421407
	20	7507.569	0.86882561
	50	7035.326	0.81417452
72h	NT	5386.912	1
	10	10644.711	1.97603209
	20	9535.347	1.77009519
	50	11289.004	2.0956355

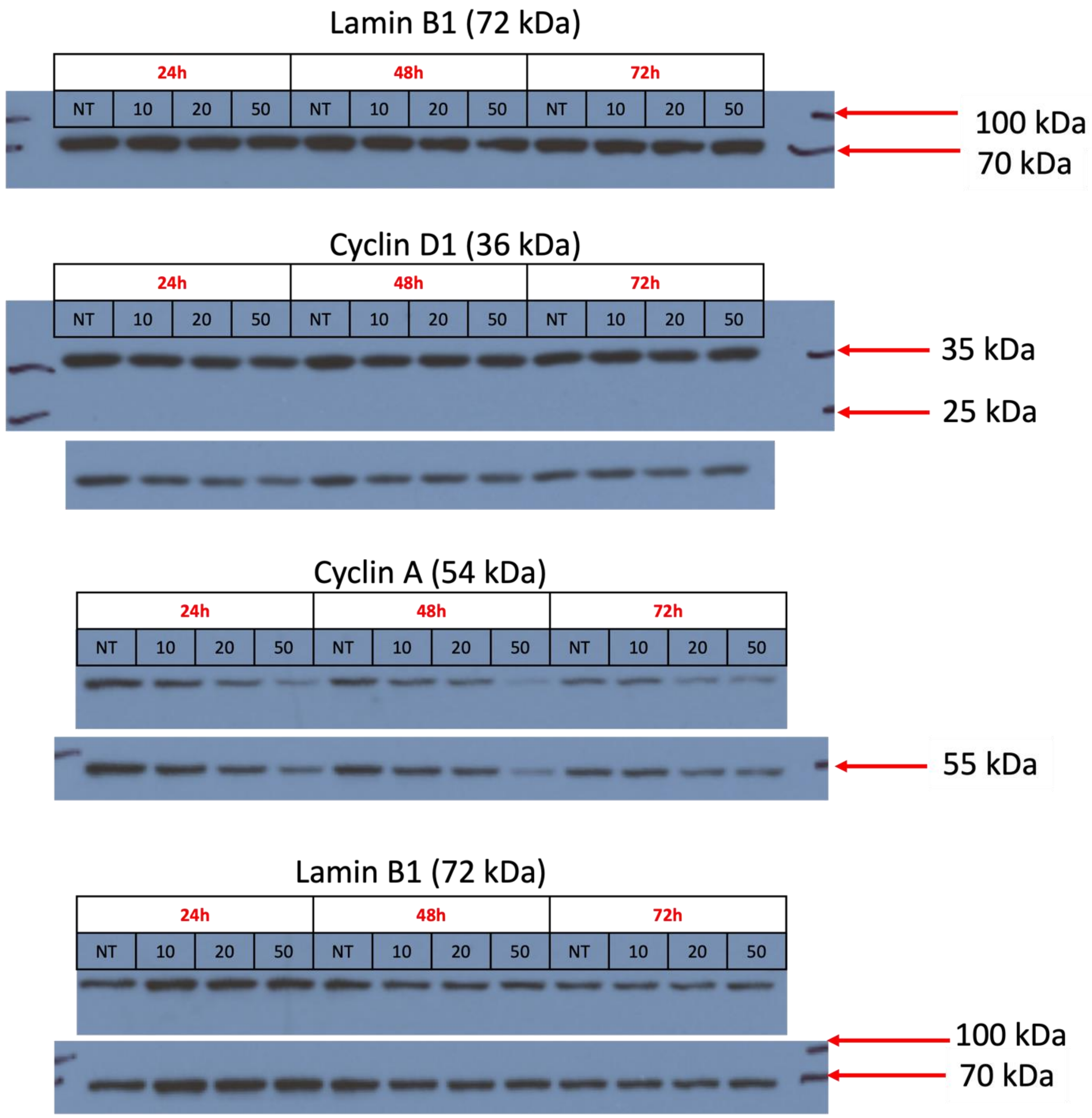
Lamin B1 (72 kDa)
24h
48h
72h
NT 10 20 50 NT 10 20 50 NT 10 20 50
100 kDa
70 kDa
Cyclin D1 (36 kDa)
24h
48h
72h
NT 10 20 50 NT 10 20 50 NT 10 20 50
35 kDa
25 kDa
Cyclin A (54 kDa)
24h
48h
72h
NT 10 20 50 NT 10 20 50 NT 10 20 50
55 kDa
Lamin B1 (72 kDa)
24h
48h
72h
NT 10 20 50 NT 10 20 50 NT 10 20 50
100 kDa
70 kDa

	Cyclin D1			normalized to lamin B1
24h	NT	8661.933	1	1
	10	5658.154	0.65322071	0.549761595
	20	4425.276	0.51088781	0.644875775
	50	3251.619	0.37539184	0.485000026
48h	NT	7154.104	1	1
	10	4774.154	0.66733081	0.72426685
	20	5348.447	0.74760543	1.311855507
	50	4344.74	0.60730736	1.271753148
72h	NT	5258.447	1	1
	10	5499.861	1.04590975	1.159733272
	20	3695.205	0.70271793	1.044944397
	50	4638.154	0.88203875	0.860293397

	lamin B1 for cyclin D1		
24h	NT	15280.217	1
	10	14976.439	0.98011952
	20	13354.024	0.87394204
	50	16032.501	1.04923255
48h	NT	14352.823	1
	10	12973.51	0.90389953
	20	11880.024	0.82771341
	50	10805.368	0.75283921
72h	NT	12452.246	1
	10	12690.731	1.01915197
	20	11657.489	0.93617561
	50	10829.167	0.86965572

	Cyclin A			normalized to lamin B1
24h	NT	7878.033	1	1
	10	4616.548	0.58600262	0.38940132
	20	2412.598	0.3062437	0.22409677
	50	889.941	0.11296487	0.08743681
48h	NT	4734.083	1	1
	10	2723.598	0.57531691	0.81761253
	20	1980.184	0.41828248	0.59591744
	50	311.678	0.06583704	0.0942296
72h	NT	2259.891	1	1
	10	2232.719	0.98797641	1.11925661
	20	831.406	0.3678965	0.47447439
	50	738.527	0.32679762	0.34071388

	lamin B1 for cyclin A		
24h	NT	6429.619	1
	10	9675.811	1.50488093
	20	8786.518	1.366569
	50	8306.811	1.29196007
48h	NT	7129.447	1
	10	5016.669	0.70365472
	20	5004.255	0.70191349
	50	4981.255	0.69868743
72h	NT	4039.719	1
	10	3565.891	0.88270768
	20	3132.305	0.77537695
	50	3874.719	0.95915557

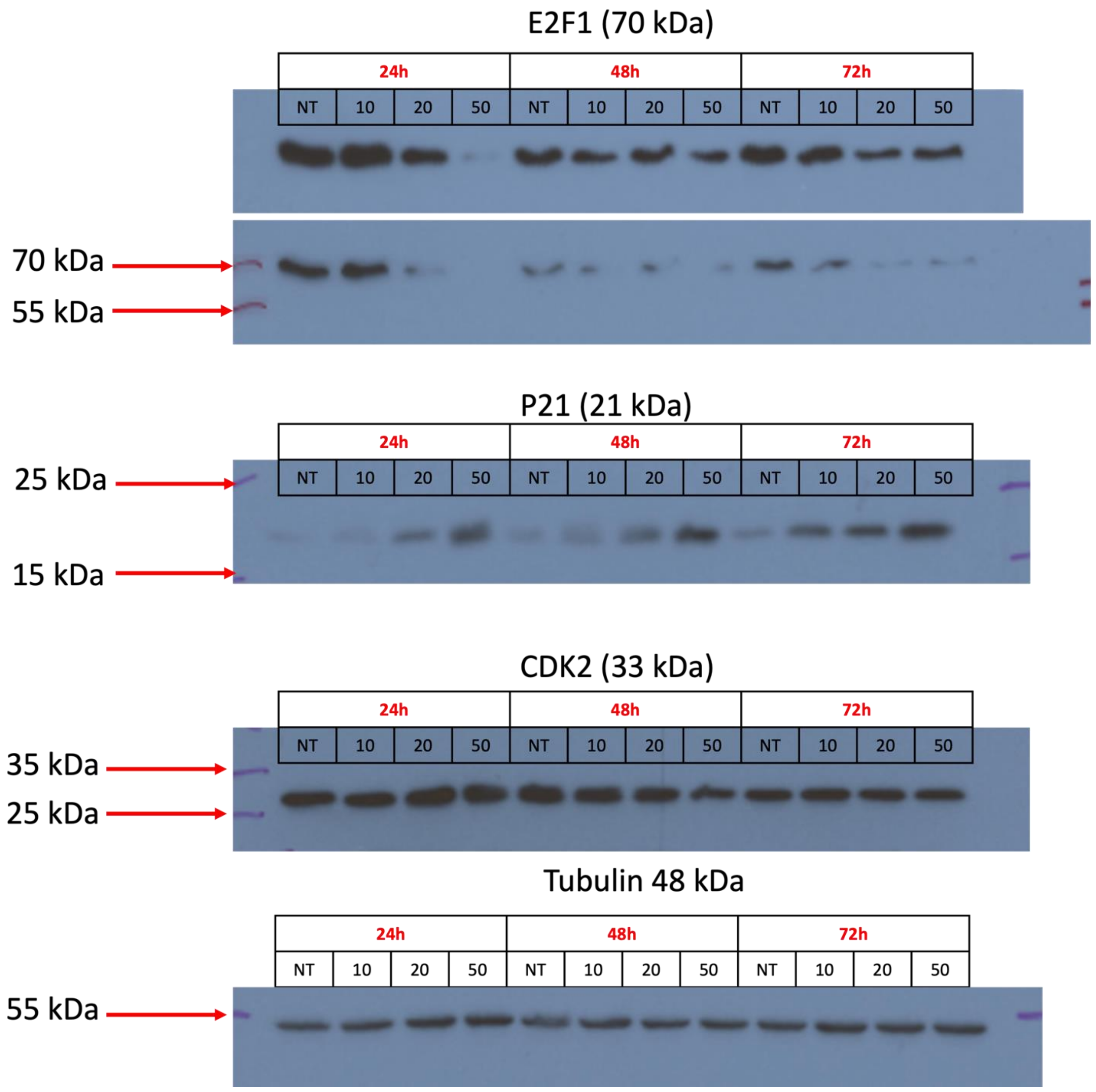
E2F1 (70 kDa)
24h
48h
72h
NT
10
20
50
70 kDa
55 kDa
P21 (21 kDa)
25 kDa
15 kDa
CDK2 (33 kDa)
35 kDa
25 kDa
Tubulin 48 kDa
55 kDa

	P21			normalized to Tubulin
24h	NT	568.849	1	1
	10	875.678	1.53938567	1.53938567
	20	39776.765	69.9249977	69.9249977
	50	39800.869	69.967371	69.967371
48h	NT	1232.163	1	1
	10	39767.246	32.2743387	32.2743387
	20	39788.735	32.2917788	32.2917788
	50	39797.555	32.2989369	32.2989369
72h	NT	1537.548	1	1
	10	5317.539	3.45845398	3.45845398
	20	7211.66	4.69036414	4.69036414
	50	13522.631	8.79493258	8.79493258

	Tubulin for P21, E2F1, CDK2		
24h	NT	3298.326	1
	10	5074.347	1.53846133
	20	6508.882	1.97338953
	50	7618.468	2.30979836
48h	NT	5834.832	1
	10	4410.468	0.75588603
	20	3277.861	0.5617747
	50	2976.447	0.510117
72h	NT	3007.276	1
	10	4596.054	1.52831134
	20	4025.761	1.3386736
	50	4704.175	1.56426447

	E2F1			normalized to Tubulin
24h	NT	19750.459	1	1
	10	20130.51	1.01924264	0.66250781
	20	10673.539	0.54041979	0.27385358
	50	156.192	0.00790827	0.00342379
48h	NT	10515.368	1	1
	10	6797.811	0.64646439	0.85524056
	20	8488.368	0.80723452	1.4369364
	50	5014.569	0.47688003	0.93484442
72h	NT	13229.61	1	1
	10	10446.489	0.7896294	0.51666789
	20	5531.276	0.41809819	0.31232273
	50	7584.882	0.5733262	0.36651488

	CDK2			normalized to Tubulin
24h	NT	14661.338	1	1
	10	13936.681	0.95057361	0.61787293
	20	16696.752	1.13882867	0.57709269
	50	14046.095	0.95803637	0.41477056
48h	NT	14663.146	1	1
	10	13404.024	0.91413016	1.20934919
	20	11846.489	0.80790909	1.4381372
	50	8214.125	0.56018845	1.09815679
72h	NT	10730.075	1	1
	10	11830.782	1.10258148	0.72143774
	20	10068.832	0.9383748	0.70097356
	50	9654.589	0.89976901	0.57520261

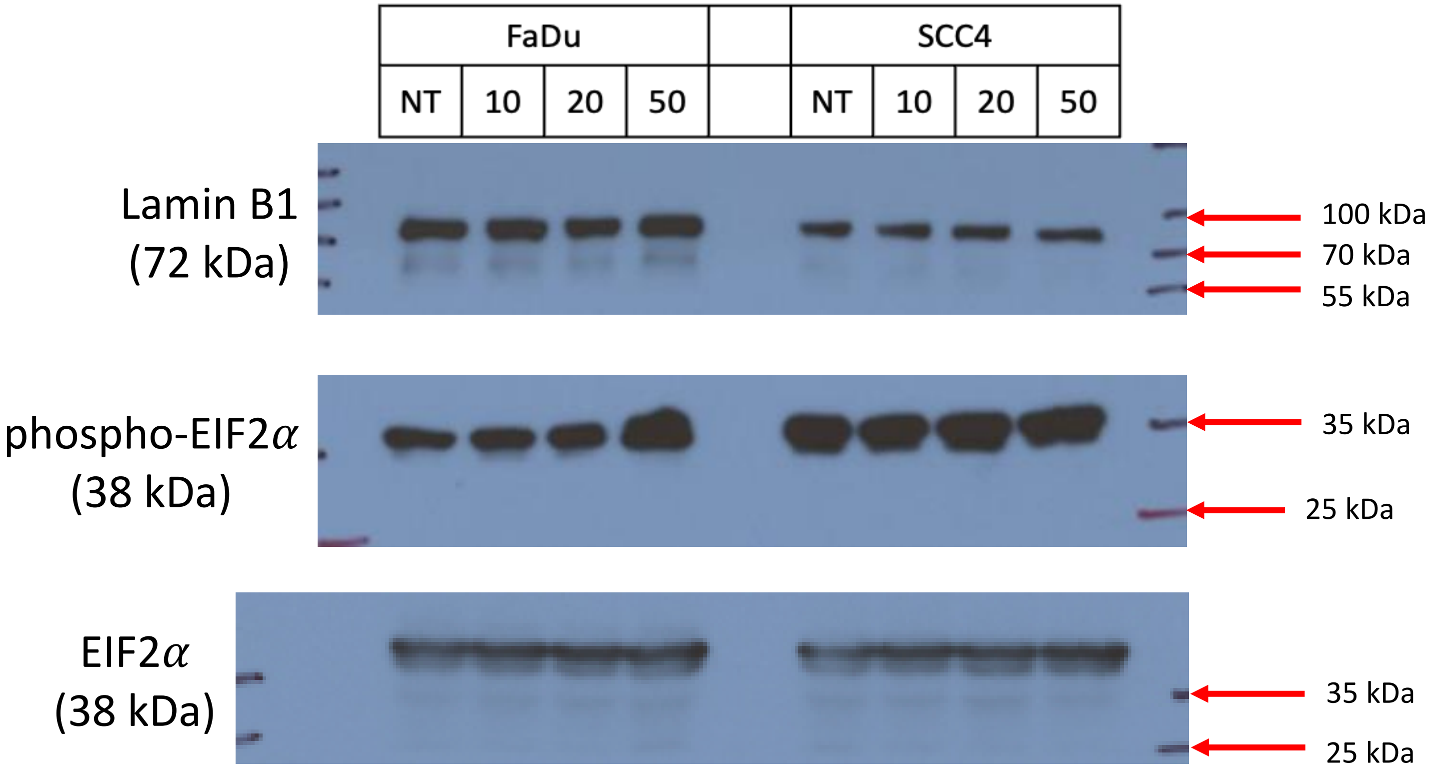
FaDu
SCC4
NT
10
20
50
NT
10
20
50
Lamin B1
(72 kDa)
100 kDa
70 kDa
55 kDa
phospho-EIF2α
(38 kDa)
35 kDa
25 kDa
EIF2α
(38 kDa)
35 kDa
25 kDa

12h		Lamin B1	
			normalized
FaDu	NT	4386.527	1
	10	4093.648	0.93323214
	20	3269.87	0.74543483
	50	4419.406	1.00749545
SCC4	NT	3357.577	1
	10	3429.698	1.02148007
	20	3771.456	1.12326717
	50	3665.456	1.09169678

12h		p- eIF2a		
			normalized	norm to Lamin B1
FaDu	NT	5042.355	1	1
	10	5188.598	1.02900292	1.102622667
	20	5502.426	1.09124129	1.463898994
	50	9134.447	1.81154381	1.798066492
SCC4	NT	8344.083	1	1
	10	8448.912	1.01256327	0.991270706
	20	9450.447	1.13259264	1.008302099
	50	10076.912	1.20767159	1.106233538

12h		eIF2a		
			normalized	norm to Lamin B1
FaDu	NT	6696.669	1	1
	10	5762.376	0.86048392	0.922047023
	20	5712.205	0.85299199	1.144287811
	50	6539.983	0.9766024	0.969336781
SCC4	NT	6874.811	1	1
	10	5759.619	0.8377858	0.820168515
	20	6414.912	0.93310376	0.830705098
	50	7347.79	1.06879884	0.979025363
